# Editorial: Cardiovascular comorbidities in inflammatory rheumatic diseases

**DOI:** 10.3389/fmed.2025.1724208

**Published:** 2025-11-17

**Authors:** Konstantinos Triantafyllias, Matteo Colina, Durga Prasanna Misra

**Affiliations:** 1 Department of Rheumatology, Acute Rheumatology Center, Bad Kreuznach, Germany; 2 Department of Internal Medicine I, Division of Rheumatology and Clinical Immunology, University Medical Center of the Johannes Gutenberg University Mainz, Mainz, Germany; 3 Service of Rheumatology, Department of Medicine and Oncology, Ospedale Santa Maria della Scaletta, Imola, Bologna, Italy; 4 Department of Clinical Immunology and Rheumatology, Sanjay Gandhi Postgraduate Institute of Medical Sciences (SGPGIMS), Lucknow, India

**Keywords:** cardiovascular disease, cerebrovascular disease, rheumatoid arthritis, psoriatic arthritis, giant cell arteritis, gout, Sjögren's disease (SD)

The survival in patients with autoimmune rheumatic diseases (ARDs) has markedly improved with the advent of better therapies, including biologic and targeted synthetic disease-modifying antirheumatic drugs (DMARDs), over the past two decades. In this context, the identification and management of comorbidities is increasingly recognized as a critical intervention toward improving the overall quality of life and prolonging survival further in these diseases. Cardiovascular (CV) comorbidity is a major cause of morbidity and mortality overall, and specifically in the context of ARDs (1, 2). The inflammatory origins of atherosclerosis are now prime targets for interventions with therapies such as canakinumab and ziltevikimab, having shown beneficial effects for CV risk (CVR) reduction in clinical trials (3, 4). CVR is uniformly increased in the ARDs, whether it be systemic lupus erythematosus (SLE), rheumatoid arthritis (RA), Sjögren's disease, systemic sclerosis, or systemic vasculitis (5–8). The risk factors driving CV comorbidity can be classified as disease-related (e.g., antiphospholipid antibodies), treatment-related (e.g., tofacitinib or glucocorticoids), or traditional risk factors such as smoking or diabetes mellitus whose prevalence is also higher in patients with rheumatic diseases (5). Imaging to detect subclinical atherosclerosis (ultrasound, coronary artery calcium detected using computed tomography, fluorodeoxyglucose positron emission tomography) or subclinical cardiac pathology [cardiac magnetic resonance imaging (MRI)] is also increasingly being used to diagnose early and stratify CV disease in rheumatic diseases (5).

In this article collection, original research and review articles provide novel insights into CV risk, disease mechanisms, and biomarkers across a spectrum of inflammatory rheumatic diseases. A Norwegian registry study of 1,821 RA patients diagnosed from 1972 to 2013 found an overall decline in stroke rates over time (Alsing et al.). However, men diagnosed after 2007 continued to have an excess stroke risk, whereas women did not, highlighting sex-specific differences in this context. In 120 RA patients undergoing coronary angiography, a nomogram combining LDL-C, Th17 cells, IL-17, and traditional CVR factors accurately predicted obstructive coronary artery disease (AUC = 0.974 in training and 0.896 in validation cohorts) (Wang et al.). Elevated CRP, Th17, and IL-17 levels in affected patients may further support the strong link between immune dysregulation and increased CVR in RA. In two studies examining CV biomarkers in RA, both inflammatory and renal indicators were shown to have important prognostic value (Liu et al.; Bin et al.). The first, involving 1,314 RA patients, found that elevated CBC-derived markers (systemic inflammatory response index, neutrophil-to-lymphocyte ratio, and monocyte-to-lymphocyte ratio) were independently associated with higher all-cause mortality (Liu et al.). The second, including 1,363 RA patients, identified the urine albumin-to-creatinine ratio as an independent predictor of all-cause and CV mortality, outperforming estimated glomerular filtration rate (Bin et al.). In a multi-ethnic cohort of 276 RA patients, the Haptoglobin (Hp) 2-2 genotype was independently associated with an increased risk of CV disease. According to the authors, the Hp 2-2 genotype could thus serve as a potential biomarker for improved CVR prediction in RA (Xu et al.).

The included review works in this Research Topic cover a broad spectrum of topics, ranging from CV imaging biomarkers to the interactions between immunosuppressive therapies and CVR. For instance, Sturm et al. analyzed the diagnostic value of ocular vascular markers (e.g., retinal vessel analysis, Optical Coherence Tomography Angiography, retrobulbar color Doppler) in patients with ARDs (Sturm et al.). Key findings included retinal arterial narrowing, venular widening, reduced capillary density, and ophthalmic vessel abnormalities, which were partially linked to systemic inflammation and traditional CVR factors. Moreover, Vitale et al. explored the role of cardiac MRI in systemic sclerosis, providing a comprehensive evaluation of its capabilities. The study highlighted MRI's ability to detect key cardiac abnormalities, including myocardial inflammation, fibrosis, microvascular dysfunction, and right ventricular strain, while also discussing its potential value as a prognostic CV tool (Vitale et al.). Heart failure with preserved ejection fraction (HFpEF) is common, and patients with ARDs face a higher risk due to inflammation, oxidative stress, endothelial dysfunction, and metabolic disturbances (9). The work of Xu et al. highlights the H2FPEF score for early risk assessment and supports interventions, such as SGLT2 inhibitors, to target disease-specific pathways and prevent HFpEF progression (Xu et al.,”Autoimmune inflammation as a key risk factor for heart failure with preserved ejection fraction (HFpEF). The different types of inflammation driving to HFpEF”; in press). In a further review focusing on the IL-6 receptor–targeting monoclonal antibody tocilizumab, the drug demonstrated sustained efficacy and a favorable safety profile in RA, with no increased CVR compared to other RA therapies. These findings reinforce tocilizumab as a key treatment option, particularly for patients with an inadequate response to methotrexate or TNF-inhibitors (Parisi et al.). The link of psoriasis to a significantly increased risk of CV disease, driven by systemic inflammation and a high prevalence of cardiometabolic comorbidities, is well-established (10). As highlighted by Barbarroja et al., apremilast provides a dual-action approach by targeting both inflammation and metabolic dysfunction, offering potential to reduce CVR and improve overall health in these patients (Barbarroja et al.). In a large retrospective study of 2,249 patients with giant cell arteritis and 3,906 patients with polymyalgia rheumatica, use of the antidepressants venlafaxine and sertraline was associated with a higher risk of CV events compared to nonusers (Zhang et al.). These findings, confirmed by both multivariate logistic regression and Cox regression analyses, suggest that the use of selective serotonin reuptake inhibitors and serotonin-norepinephrine reuptake inhibitors may increase CVR in this patient population. A retrospective study of 292 gout patients found that tophi, higher power Doppler signals, and frequent flares independently predicted carotid plaque vulnerability (Fang et al.). A random forest model combining these gout-specific factors with traditional CVR showed excellent predictive accuracy (C-index = 0.997), highlighting the role of crystal-driven inflammation in vascular injury and the value of integrated risk assessment. Interestingly, in a retrospective study of 49 Sjögren's-disease patients and 27 matched controls, no differences in endothelial function, measured by reactive hyperaemia index using peripheral arterial tonometry (EndoPAT^®^), were observed between the groups. The only factor associated with impaired endothelial function was higher body mass index. The authors concluded that EndoPAT may not be sensitive for detecting Sjögren's-specific vascular changes, highlighting the need for alternative markers in this specific patient group (Tapken et al.).

To summarize, this article collection highlights the increased risk of CV disease and potential biomarkers in psoriatic arthritis, RA, Sjögren's disease, systemic sclerosis and Giant Cell Arteritis. Articles also delineate the role of cardiac MRI and ultrasound for detecting subclinical CV pathology in inflammatory rheumatic diseases. The role of therapies such as apremilast on modulating CVR has also been explored. The editors hope this article collection serves as a nidus to promote further translational research on CVR in rheumatic diseases.
